# Axisymmetric acoustophoresis for paper pulp concentration

**DOI:** 10.1016/j.ultsonch.2021.105822

**Published:** 2021-11-06

**Authors:** Romain Le Magueresse, Tamara Krpic, Maxime Bilodeau, Robert Schiavi, Pierre Gelinas, Nicolas Quaegebeur

**Affiliations:** a GAUS – Dept Génie Mécanique, Université de Sherbrooke, Sherbrooke QC J1K2R1, Canada; b Valmet - 79 Warren Street, Glens Falls NY 12801, USA

**Keywords:** Acoustophoresis, Papermaking, Fiber suspension flow, Pulp flow simulation, Paper pulp concentration

## Abstract

In pulp and paper mills, mechanical processes such as screening and washing are commonly used to remove accumulated solid suspensions and concentrate the pulp. For environmental reasons and to optimize paper production, an emerging challenge is to develop alternative methods to concentrate paper pulp between 3 % and 6 % consistency for which the mixed pulp-water flow is complex. Among the proposed solutions in the literature, solutions based on acoustic levitation, also referred as acoustophoresis, of low-consistency pulp have been demonstrated as a potential solution for efficient pulp concentration and water recirculation. However, no sensitivity analysis on the ultrasound and physical parameters was proposed, limiting the extension to a realistic application. Thus, this paper presents a numerical modeling of acoustophoresis for pulp flow concentration in a pipe. For this purpose, the pulp flow is defined as a pseudo-homogenous fluid with a turbulent Low Re k- ∊ formalism, and the pulp particles are considered spherical and deflected by two acoustic forces, namely the acoustic radiation force and the Stokes drag force, both induced by an ultrasound wave generated along the walls of a circular pipe. The combined action of these two forces in the pulp flow enables to concentrate the particles at the center of the pipe. The influences of particle size and mechanical properties, fluid properties and ultrasound parameters are analyzed within a parametric study to optimize the particle deflection and the pulp concentration. The experimental feasibility of the industrial use of acoustophoresis for the concentration of paper pulp is demonstrated with a concentration gain up to 15 %.

## Highlights

• Numerical simulations of acoustophoresis in pulp flow is conducted.
• Parametric study of fiber characteristics, pulp flow regimes and acoustic parameters are conducted.
• A feasibility study of industrial acoustophoresis for pulp concentration is performed.
• A potential concentration gain of 15% is obtained experimentally.

## 1 Introduction

In pulp and paper mills, mechanical processes [1] such as screening and washing are commonly used to remove accumulated solid suspensions and concentrate the pulp. Screening of the pulp is a step that separates, with the help of sieves, all the wood residues that are not cellulosic fibres: knots or debris. The resulting pulp consistency is below 3 %. After this screening step, pulp washing is used to recover the cooking chemicals and to increase the consistency from 6 % to 30 %. With regards to paper production efficiency and environmental reasons, an emerging challenge is to develop new paper pulp concentration methods. Indeed, 3 % to 6 % consistency in complex mixed pulp-water flow (non-Newtonian fluid, existence of a transition phase between turbulent and laminar flow) is needed [2], [3].

Indeed, the flow of pulp fiber suspension in a pipe is characterized by three distinct regimes. At low velocities (typically below 0.1 m/s), the flow exhibits the shape of a fiber-plug, for which shear occurs in a boundary layer along the wall and the velocity profile is uniform in the bulk region. For medium velocities (typically around 0.5 m/s) a transition zone occurs where the central plug is surrounded by a ring where the water-fiber mixture is turbulent. At high speed (above 1 m/s typically) the entire fluid is turbulent and can be considered as water [2].

Among the proposed solutions in the literature, the experimental work of H. Brodeur based on acoustic levitation, also referred as acoustophoresis, of low-consistency pulp [4], [5], [6] has demonstrated a potential solution for efficient pulp concentration and water recirculation. The objective of this technique is to remotely act on the pulp fibers using a high-power ultrasonic field that will induce a displacement of the fibers and surrounding fluid through the combined effect of the radiation acoustic force and the drag force. However, these experimental studies did not lead to an industrial solution since the pulp consistencies were chosen below 1 % and thus not aligned with industrial requirements. Moreover, since the published work was mostly experimental, no sensitivity analysis on the ultrasound and physical parameters was proposed, limiting the extension to a realistic application.

In the last decade, the numerical modeling of acoustophoresis has received much attention for micro-fluidic systems. Indeed, today there are many applications of the use of acoustic levitation in static microfluidic environments for biological and pharmaceutical applications [7], [8], [9]. The influence of particle size and mechanical properties, fluid properties and ultrasound parameters has been extensively studied using Finite Element Models (FEM) for micro-sized applications. However, to the best of our knowledge, no extension to macro-sized or industrial systems in the presence of a mean flow has been proposed in the literature. Thus, in order to investigate the feasibility and the extension of acoustophoresis for high-consistency pulp, the extension of existing numerical models developed for microfluidic environments is proposed in the present study for a pulp flow in a pipe in the presence of a mean flow.

In Section 2, the governing equations of acoustophoresis are derived in the case of a fiber-pulp mixture in the presence of a mean flow. Subsequently, Section 3 proposes a numerical implementation of this model. The results are presented in Section 4, including a parametric study in order to optimize the deviation of paper pulp particles. Finally, Section 5 presents the experimental results obtained at low velocity.

## 2 Modeling acoustophoresis in a pulp flow

### 2.1 Geometry of interest

**Fig. 1:**
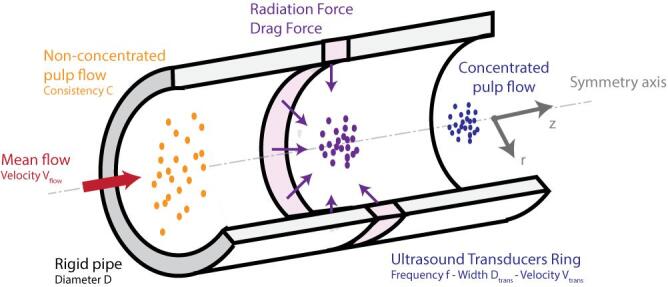
Schematics of the pulp concentration using acoustophoresis.

In the present study, we consider a cylindrical configuration of acoustophoresis for which the aim is to concentrate the fibers towards the center of a cylindrical pipe using an axisymmetric ring of transducers as presented in Fig. 1.

The fluid is defined by its velocity u , pressure p and density ρ . Each of these variables is defined as the sum of the average value ($u_{0}$ , $p_{0}$ , $\rho_{0}$) induced by the mean flow and the first ($u_{1},p_{1},\rho_{1}$) and second order ($u_{2},p_{2},\rho_{2}$) terms:

(1) $$u\left(r,z,t\right)=u_{0}\left(r\right)+u_{1}\left(r,z,t\right)+u_{2}\left(r,z\right)$$

(2) $$p\left(r,z,t\right)=p_{0}\left(r\right)+p_{1}\left(r,z,t\right)+p_{2}\left(r,z\right)$$

(3) $$\rho \left(r,z,t\right)=\rho_{0}\left(r\right)+\rho_{1}\left(r,z,t\right)+\rho_{2}\left(r,z\right)$$

The terms ($u_{0}$ , $p_{0}$ , $\rho_{0}$) correspond to the average flow of pulp in the pipe and only depend on the radial position r since the mean flow is assumed to be established and thus independent of the z position. This flow is detailed in section 2.1. The first order disturbance terms ($u_{1},p_{1},\rho_{1}$) presented in section 2.2 corresponds to the harmonic acoustic fields induced by the transducers at the angular frequency ω. These terms allow the derivation of the acoustic radiation force. The second order terms ($u_{2},p_{2},\rho_{2}$) is related to the acoustic streaming induced by the first order terms and is thus time-independent. Those terms introduced in section 2.3, are responsible for the drag force.

### 2.2 Pulp flow model ($u_{0},p_{0},\rho_{0})$

In our study, the fluid is considered as pseudo-homogeneous, where interactions of particles with each other are neglected, such that all particles are independent. There are several ways to numerically model the pulp flow [10] depending on the velocity and consistency regimes. The first regime that corresponds to the plug flow can be modeled by a laminar flow by imposing sliding conditions on the walls [2]. The second regime, also referred as the transition regime, is more difficult to model. In this case, a laminar flow could be used for the central part and a turbulent flow in the lateral part. The third regime corresponding to high velocities can be considered as turbulent, and is commonly modeled using k-∊ formalism [11] or the LowRek-∊ [12], [13]. For the k-∊ model, the flow is not calculated in the buffer region close to the walls, since wall functions are used in order to avoid modeling the boundary gradient at the walls. The LowRek-∊ model can be seen as an extension of the k-∊ model since it doesn’t require wall approximations and the flow is solved everywhere, requiring a very high mesh resolutions, and thus a high computational cost.

In the present study, the LowRek-∊ formalism is used for computing the mean flow and allows modeling the plug and turbulent phases accurately. In the present study, the AKN (Abe, Kondoh, and Nagano [14]) formalism is chosen to model the pulp and can be expressed as the system of nonlinear partial derivative equations derived in [15] for the axial and radial coordinates.

In addition, the paper pulp-water mixture is considered as a non-Newtonian fluid whose viscosity μ is not constant and depends on different parameters. A simple model is to consider the viscosity of the pulp flow as the ratio between stress and shear rate [16]. A more complete model was then validated by Cotas [15], in which the pulp concentration and shear rate are taken into account. This model has been experimentally validated for different pulp types and is given by:

(4) $$\mu =K^{\text{'}}C^{\alpha }{\left(\dot{\gamma }\right)}^{\beta C}$$

where $K^{\text{'}},\alpha ,\beta$ are defined for both eucalyptus pulp (Hardwood pulp) and pine pulp (Softwood pulp) in [16] in order to obtain the viscosity for each pulp type. Those viscosity parameters are then used for the calculation of the LowRek-∊ model and first order terms calculations.

### 2.3 First order terms ($u_{1},p_{1},\rho_{1})$

In this section, the fist order terms ($u_{1},p_{1},\rho_{1}$) are considered. For this purpose, the models developed for micro-fluidic environments [17] are introduced and adapted for the present study. The acoustic field induced by the transducer ring is computed assuming a harmonic regime at angular frequency ω . To derive the governing equations, the heat transfer equation is first considered for temperature $T_{1}$ and combined with the continuity equation and the Navier-Stokes dynamic equation, assuming the fluid to be incompressible. Those fields are considered as first-order perturbations with respect to the mean fields induced by the pulp flow and since the pulp flow speed is very low compared to the acoustic wave speed, both fields are decoupled, and the transport terms are omitted [17] :

(5.a) $$\frac{\partial T_{1}}{\partial t}=D_{\mathrm{th}}\nabla^{2}T_{1}+\alpha \frac{T_{0}}{\rho_{0}C_{p}}\frac{\partial p_{1}}{\partial t}$$

(5.b) $$\frac{\partial \rho_{1}}{\partial t}+\frac{1}{\gamma \kappa }\nabla ∙u_{1}=\frac{\alpha }{\gamma \kappa }\frac{\partial T_{1}}{\partial t}$$

(5.c) $$\rho_{0}\left(\frac{\partial u_{1}}{\partial t}+u_{1}∙\nabla u_{1}\right)=-\nabla p_{1}+\mu \nabla^{2}u_{1}+\left(\beta \mu +\mu_{B}\right)\nabla \left(\nabla ∙u_{1}\right)$$

with β the viscosity ratio which is 1/3 for a simple liquid, $D_{\mathrm{th}}$ the thermal diffusivity of the fluid, γ the specific heat rate, κ the isentropic compressibility and α the thermal expansion coefficient.

As noted in [17], two distinct boundary layers are obtained for the variables $u_{1}$ and $T_{1}$ . They are referred to as the viscous and thermal boundary layers lengths, under harmonic assumption at angular frequency ω , and with $\nu =\frac{\mu }{\rho_{0}}$ the cinematic viscosity:

(6.a) $$\delta_{th}=\frac{\sqrt{2D_{th}}}{\omega }$$

(6.b) $$\delta =\sqrt{\frac{2\nu }{\omega }}$$

These two penetration lengths define the thickness of the boundary layers at the walls, and thus the limit for the mesh when solving the first order terms.

Moreover, the acoustic attenuation $\alpha_{\mathrm{acous}}$ in pulp is considered in the model. For this purpose, the formalism proposed by McFarlan [18] and Lofqvist [19] is used in order to estimate the value of acoustic attenuation based on the bulk viscosity $\mu_{B}$ . This attenuation is thus dependent of the pulp consistency and of the frequency used.

### 2.4 Second order terms ($u_{2},p_{2},\rho_{2})$

The second order terms are now considered to obtain the induced acoustic stream. In this case, the coupling between the temperature field $T_{2}$ and the fields $p_{2}$ and $u_{2}$ is neglected, and the second order continuity equation as well as Navier Stokes' equation are used:

(8.a) $$\frac{\partial \rho_{2}}{\partial t}+\rho_{0}\nabla ∙u_{2}+\nabla \left(\rho_{1}u_{1}\right)=0$$

(8.b) $$\rho_{0}\frac{\partial u_{2}}{\partial t}+\rho_{1}\frac{\partial u_{1}}{\partial t}+\rho_{0}\left(u_{1}∙\nabla \right)u_{1}=-\nabla p_{2}+\mu \nabla^{2}u_{2}+\beta \mu \nabla \left(\nabla ∙u_{2}\right)$$

After time-averaging those two equations over a period of oscillation denoted <⋯> , the following equations are obtained:

(9.a) $$\rho_{0}\nabla ∙<u_{2}>=-\nabla ∙<\rho_{1}u_{1}>$$

(9.b) $$<\rho_{1}\frac{\partial u_{1}}{\partial t}>+\rho_{0}<\left(u_{1}∙\nabla \right)u_{1}>=\mu \nabla^{2}<u_{2}>+\beta \mu \nabla \left(\nabla ∙<u_{2}>\right)-<\nabla p_{2}>$$

Those two equations allow deriving the average particle motion over a period of oscillation and will be used to derive the forces in action, i.e. radiation and drag forces.

### 2.5 Forces in action

In order to model acoustophoresis, different fibers are introduced in the model defined by their respective position. For the sake of simplicity, the fibers are modeled as spherical particles of radius a and density $\rho_{p}$ instead of as cylindrical particles [20]. This allows neglecting the degree of freedom in rotation [20] of each fiber. In addition, any interactions between particles and the effect of gravity are neglected. Consequently, the radiation and drag forces characteristic of acoustophoresis can now be derived. The radiation force on a spherical particle of radius a subjected to the pressure field is defined as follows [21]:

(10) $$F_{\mathrm{rad}}=-\pi a^{3}\left(\frac{2\kappa_{0}}{3}Re\left[f_{1,rad}^{*}p_{1}^{*}\nabla p_{1}\right]-\rho_{0}Re\left[f_{2,rad}^{*}u_{1}^{*}.\nabla u_{1}\right]\right)$$

with $\kappa_{0}=\frac{1}{\rho_{0}c_{0}^{2}}$ the fluid compressibility, $f_{1,rad}\left(\overset{\;}{\kappa }\right)=1-\frac{\kappa_{p}}{\kappa_{0}}$ ; $f_{2,rad}\left(\overset{\;}{\rho },\overset{\;}{\delta }\right)=\frac{2\left(1-\Gamma \left(\overset{\;}{\delta }\right)\right)\left(\overset{\;}{\rho }-1\right)}{2\overset{\;}{\rho }+1-3\Gamma \left(\overset{\;}{\delta }\right)}$ with $\overset{\;}{\rho }=\frac{\rho_{p}}{\rho_{0}}$ ; $\Gamma \left(\overset{\;}{\delta }\right)=-\frac{3}{2}\left(1+i\left(1+\overset{\;}{\delta }\right)\right)\overset{\;}{\delta }$ , with $\overset{\;}{\delta }=\frac{\delta }{a}$ .The time-averaged Stokes drag force on a spherical particle of radius a , moving with velocity u in a fluid having a mean flow $u_{0}$ and a streaming velocity $u_{2}$ **,** is adapted from the expression [17]:

(11) $$F_{\mathrm{drag}}=6\pi \mu a\left(u_{0}+<u_{2}>-u\right)$$

These two forces act on each particle simultaneously such that the resulting movement of particles occurs through the combined effect of the radiation acoustic force and the drag force. The radiation acoustic force attracts particles into the pressure nodes of the $p_{1}$ sound field, while the drag force deflects the particles through the acoustic stream $<u_{2}>$ created by the harmonic excitation. It is therefore interesting to determine which force predominates over the other one for a given configuration or frequency.

## 3 Numerical implementation of the model

### 3.1 Simulation parameters:

In this section the numerical model and its implementation on the multiphysics software COMSOL 5.4 are presented. The studied area is an axisymmetric pipe of length L and diameter D such that a 2D numerical model is implemented in order to reduce the calculation time compared to a 3D model. The piezoelectric transducer is represented by a radiating surface of diameter $D_{\mathrm{trans}}$ with a normal vibration velocity $V_{\mathrm{trans}}$ at frequency f . For the fluid and wood particles, the parameters presented in Table 1 are used based on [22]. The effect of some parameters such as frequency f , particle radius a , density $\rho_{p}$ , fluid viscosity μ , transducer velocity $V_{\mathrm{trans}}$ , and mean fluid velocity $V_{\mathrm{flow}}$ are studied in detail during the parametric study.

**Table 1:** Parameters for the domain, particles and fluid.

Element	Parameter	Notation	Value	Reference
Pipe	Diameter [mm]	D	200	
	Length [mm]	L	600	
Transducer	Diameter [mm]	$D_{\mathrm{trans}}$	50	
	Frequency [kHz]	f	20–300	40
	Excitation velocity [$m.s^{-1}$]	$V_{\mathrm{trans}}$	0.1–2	0.5
Wood particles	Radius [μm]	a	5–1000	10
	Density [$kg.m^{-3}$]	$\rho_{p}$	700–1500	900
	Speed of sound [$m.s^{-1}$]	$c_{p}$	3300	
	Transverse speed of sound [$m.s^{-1}$]	$c_{p_{t}}$	1100	
	Poisson’s ratio	$\sigma_{p}$	0.35	
	Compressibility	$\kappa_{p}$	1.47e-10	
Fluid/Water	Consistency (%)	C	0.1–3	1.5
	Viscosity (Eucalyptus)	μ	$10^{-2.07}C^{6.97}{\left(\dot{\gamma }\right)}^{-0.26C}$	
	Viscosity (Pine)	μ	$10^{-1.12}C^{5.90}{\left(\dot{\gamma }\right)}^{-0.28C}$	
	Inflow velocity [$m.s^{-1}$]	$V_{\mathrm{flow}}$	0.01–0.5	0.05
	Density [$kg.m^{-3}$]	$\rho_{0}$	1000	
	Speed of sound [$m.s^{-1}$]	$c_{0}$	1500	
	Compressibility [$pPa^{-1}$]	$\kappa_{0}$	448	
	Thermal conductivity [$W.m^{-1}K^{-1}$]	$k_{\mathrm{th}}$	0.603	
	Specific heat capacity [$J.kg^{-1}K^{-1}$]	$C_{p}$	4180	
	Specific heat capacity ratio	γ	1.014	
	Thermal diffusivity [$m^{2}s^{-1}$]	$D_{\mathrm{th}}$	1.43e-7	
	Thermal expansion ratio [$K^{-1}$]	α	3e-6	

### 3.2 Numerical implementation

The mean flow is implemented using the *Turbulent Flow Low Re k-∊* module. The parameters are adjusted according to the model presented in the section 2 and Table 1. The boundary conditions are defined as follow: at the inlet, a normal mean velocity $V_{\mathrm{flow}}$ with turbulent flow constants as presented in section 2.1 and a turbulent intensity of 5 %, and at the outlet, the pressure is considered null. A no-slip condition is applied to the side wall. For this step, a physics-controlled mesh is used with extra fine elements. Thus, a sequence type mesh for fluid dynamics in COMSOL is implemented with triangular elements, of a maximal length of 1 mm, and a local refinement with an element size scaling factor of 0.2 around the edge of the pipe is used in order to accurately describe the boundary layer of the mean flow. This step requires 75 000 elements for a total of approximately 200 000 degrees of freedom. The nonlinear system that the Navier-Stokes (RANS) and turbulence transport equations constitute is solved using a segregated approach [23]. Thus, for each iteration in the Navier-Stokes group, two or three iterations are performed for the turbulence transport equations. The default iterative solver for the turbulence transport equations is a damped Newton method with constant damping factor. In each iteration, a linearized version of the nonlinear system is solved using Generalized Minimal RESidual method (GMRES) accelerated by Smoothed Aggregated Algebraic Multigrid (SAAMG). The default smoother is SOR Line.

For the first and second order terms, the *Thermoviscous Acoustics* and *Laminar Flow* modules are respectively used. The *Thermoviscous Acoustics* module allows calculating the first order acoustic field as proposed in [17]. This calculation is performed assuming harmonic excitation at the frequency f . For this purpose, an isothermal condition is applied to the side wall and the inlet and outlet faces are assumed to be infinite, such that a Perfectly Matched Layer (PML) of three acoustic wavelengths long is added at both ends of the pipe. The excitation terms correspond to the transducer which imposes a normal velocity. In order to avoid the discontinuities at the edge of the transducer, a spatial windowing (Hanning window) is used over a length $D_{\mathrm{trans}}$ with a maximal value of $V_{\mathrm{trans}}$ .

**Fig. 2:**
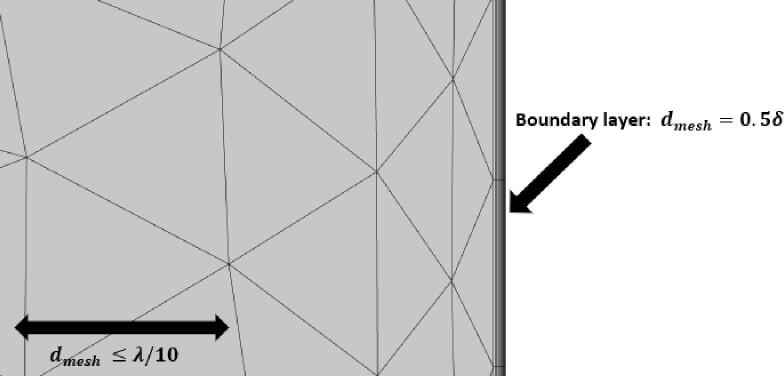
Details on the boundary layer used for computation of the first and second order terms.

Then, the second order terms are computed with the *Laminar Flow* module where the additional terms in Eq. (10.b) are added as weak contributions, as suggested in [17] . For this step, a second more refined mesh is used based on the acoustic and thermal field properties. Thus, the mesh is composed of triangular elements whose maximum size is defined as 1/10 of the wavelength λ . In addition, it is necessary to refine the mesh at the domain boundaries in order to correctly model the thermo-viscous effects close to the pipe walls. To capture them we use the built-in 10 boundary elements in COMSOL whose maximal size is defined as $d_{\mathrm{mesh}}=0.5\delta$ as indicated in Fig. 2; where δ is defined as the boundary layer size in section 2. Thus, the model contains 230 000 elements for a total number of approximately 2 000 000 degrees of freedom. The equations presented in section 2.2 and 2.3 are solved with a

PARDISO solver. Based on the first and second order terms, the acoustic radiation and drag forces are extracted at each point of the domain. The *Particle Tracing* module is then used to apply the forces involved to a set of particles injected at the inlet. These particles have the same velocity as the mean pulp flow. The effect of gravitation is neglected and so only the radiation and drag forces induced by the acoustic field are opposed to the drag force induced by the mean flow. At the inlet of the pipe, particles are equally distributed over the pipe radius and the trajectory of each particle is computed through to the outlet thereby allowing the calculation of the radial deflection for each particle.

## 4 Numerical results

### 4.1 Reference configuration

**Fig. 3:**
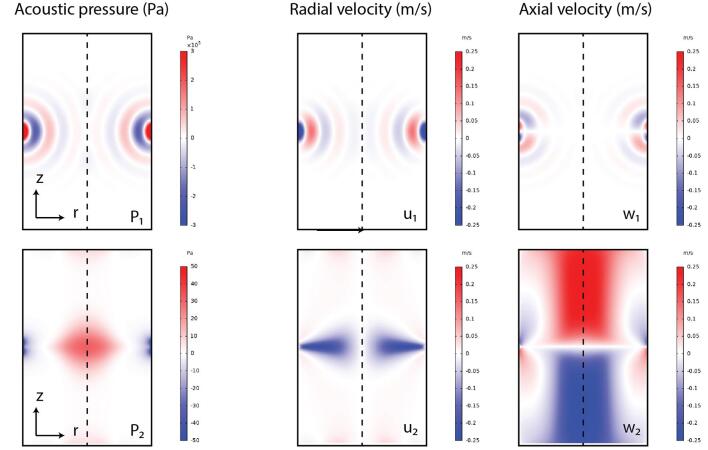
Estimated pressure field (left), radial velocity (middle) and axial velocity (right) for the first order (top) and second order terms (bottom).

In this section, a reference configuration is first considered to present the typical results and allow for determining the influence of the parameters under consideration. In this case, the reference values from Table 1 are used and determined based on experiments from the literature and preliminary experiments carried out in realistic conditions. For this purpose, a pulp consistency of 1.5 % for the eucalyptus wood is selected. Thus, the viscosity is obtained by considering the formula (4) for an inlet flow velocity of 0.1m/s . The transducer size and velocity are based on typical values obtained for Langevin transducer excited around their resonance frequency of 40 kHz. Therefore, as a result of our model presented in section 2 and 3, we obtain the first order and the second order fields in Fig. 3.

As shown in Fig. 3, the pressure and radial velocity fields $p_{1}$ and $u_{1}$ are characterized by a succession of minima and maxima and a maximal value close to the transducers due to acoustic damping. There are three of them in front of the transducer, corresponding to a resonance at the wavelength $\lambda =\frac{c}{f}=37.5mm$ , which is approximately a third of the pipe radius of 100mm , thus creating a standing wave. Moreover, the pressure maxima $p_{1}$ corresponds to the velocity minima $u_{1}$ , and vice versa, creating an acoustic radiation force that should trap the particles at the velocity nodes or antinodes depending on the acoustic contrast factor.

In contrast with the first order pressure field $p_{1}$ , the second order pressure field $p_{2}$ is maximal on the axis of symmetry of the pipe. In front of the transducers, a negative radial velocity area is observed corresponding to a zone of concentration of the pulp towards the center of the pipe. Indeed, in this zone, the radial velocity $u_{2}$ will push the particles towards the center of the pipe thanks to the drag force. The minimum value in the reference configuration is -0.25m/s . Finally, the axial velocity $w_{2}$ is presented. This velocity has a maximum absolute value of 0.25m/s . In the upper part of the pipe this velocity is positive and in the lower part this velocity is negative. This is characteristic of the acoustic stream created by the wave.

**Fig. 4:**
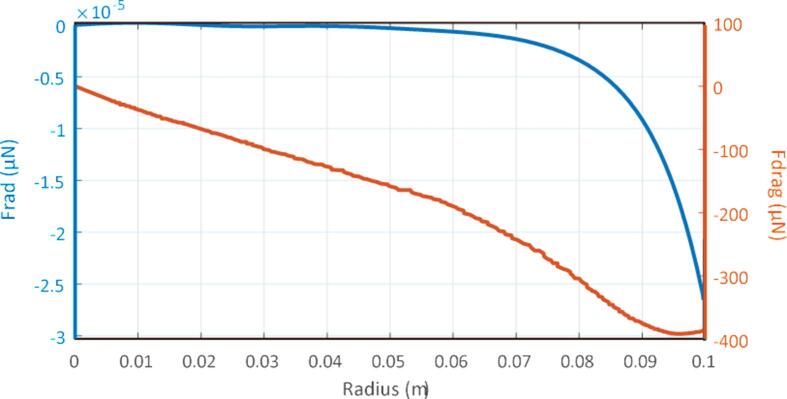
Radial acoustic radiation and drag forces in front of the transducer for z = L/2.

Thereafter, only radial velocities $u_{1}$ and $u_{2}$ are presented since they are preponderant and will be responsible for the radial compression of the pulp. Following this calculation, the acoustic radiation and drag forces in the plane in front of the transducer for z=L/2 are presented in Fig. 4.

The radial component of the acoustic radiation force has a succession of maxima and minima near the symmetry axis corresponding to the pressure nodes of the acoustic field p1 . Near the boundary of the wall, the acoustic radiation force is negative and reaches a minimum of $-2.510^{-5}\mu N$ . Wave propagation here leads to a zone of instability for the acoustic radiation force. Thus, the particles are pushed towards the center of the pipe. For the radial drag force, the pattern follows the one for the radial velocity $u_{2}$ and the central area provides a negative drag force that will concentrate the particles towards the center of the pipe. The maximum force is -400μN and is predominant. Thus, the drag force is nearly $10^{7}$ times higher than the acoustic radiation force for our application. Therefore, the acoustic streaming induced by the transducer excitation pushes the particles towards the center of the pipe (at r=0) and the particles are not attracted toward sound pressure nodes as for typical acoustic levitation applications. This can be explained by the high viscosity of the fluid and the limited size of the particles. The objective of the following section is to study the influence of the parameters involved in this model of acoustophoresis in a pulp flow in order to determine if an optimal configuration for pulp concentration exists, and determine its feasibility for industrial application.

### 4.2 Influence of the acoustic parameters

**Fig. 5:**
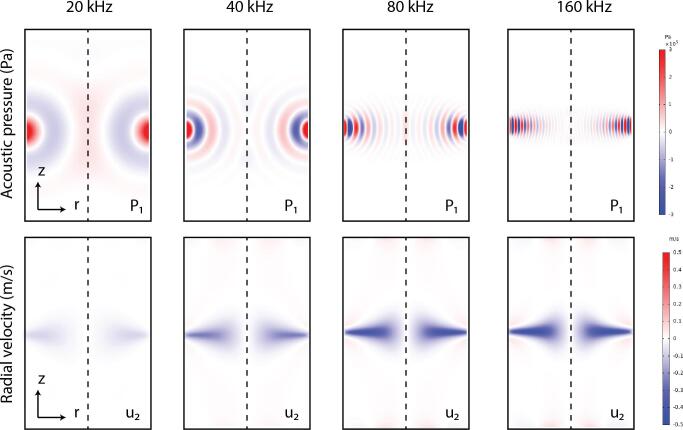
Pressure field $p_{1}$ (top); Radial drag force (bottom) obtained at different frequencies from 20 kHz to 160 kHz.

In this section, the reference parameters are used and only the transducer frequency is changed from 20 kHz to 160 kHz. The objective is to estimate the optimal frequency for our situation. The transducer chosen during the experimental phase will have a resonance frequency close to the frequency obtained from this optimal frequency. Due to the large size of our domain $\left(L=600mm\right)$ , and the frequency dependent meshing process used for the first and second order computation, it is computationally greedy to test higher frequencies. The first-order pressure fields, the radiation force, and the drag force fields are presented in Fig. 5 for each frequency.

**Fig. 6:**
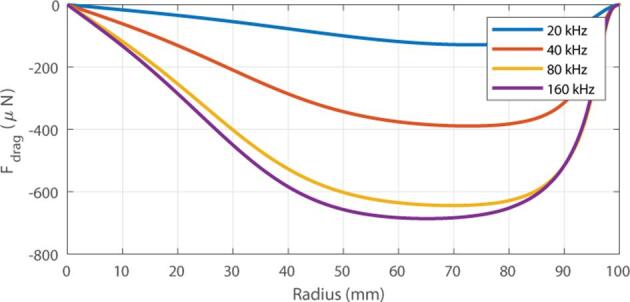
Influence of the transducer frequency on the radial drag force.

The contribution of both forces in the axis of the transducer (for z=0) is presented in Fig. 6. When the frequency is increased, the wavelength decreases, and the number of pressure nodes naturally increases. In addition, the intensity of the radiation force increases with the frequency but remains negligible. For the drag force, the same effect is observed, with an increase in the absolute value in front of the transducer from -130μN at 20 kHz to -700μN at 160 kHz. These observations confirm the experimental results of Brodeur [5] who observed that the forces are more pronounced at higher frequencies. However, a saturation effect occurs between 80 kHz and 160 kHz due to acoustic damping that limit the first order pressure field and thus the second order velocity field.

### 4.3 Influence of the particle flow properties

Particle properties

As detailed in section 3, the particles are assumed spherical with a radius a and a density $\rho_{p}$ . The radius of the particle plays a major role in the evolution of both drag and radiation forces. In practice, for pulp flow concentration, the fiber radius is not calibrated since the fiber particles tend to gather in a pulp stream to form larger particles. Thus, variations of pulp diameter from 5μm to 1mm are expected in practical cases [3]. We notice that the radius is present in both forces in action. Indeed, the drag force varies linearly with respect to the particle radius a , while the radiation force follows a cubic law, as described in Eq. (10). Thus, when considering the minimum pulp radius of 5μm , the drag force is highly dominant. However, by imagining a particle aggregate with a radius of 1mm , the acoustic radiation force becomes dominant close to the axis of the pipe and reaches up to -100μN . In this case, the particles are attracted to the pressure nodes and a mixed regime between drag and radiation is observed.

The density of the particle is of lesser importance for our study. Indeed, this value has no influence on the drag force but plays a slight role in the value of the radiation force. In Eq. (10), the term $f_{2,rad}$ depends on the density of the particle. Thus, the radiation force increases with respect to the density of the pulp and its maximal value is almost doubled when the density is increased from $900kg.m^{-3}$ to $1500kg.m^{-3}$ ; however, the order of magnitude is therefore still negligible for small particles.

Pulp flow Velocity and consistency

As presented in section 2, the pulp flow is assumed to be either laminar or turbulent depending on the inlet flow velocity and modeled with the LowRek-∊ model. The impact of the mean flow on the deviation and therefore the concentration process is thus studied in this section for inlet velocities between 0.0125 m/s and 0.2 m/s and pulp consistency of 1.5 %. The viscosity model of Eq. (4) developed for paper pulp flows is used, allowing the calculation of the fluid viscosity μ in the model.

**Fig. 7:**
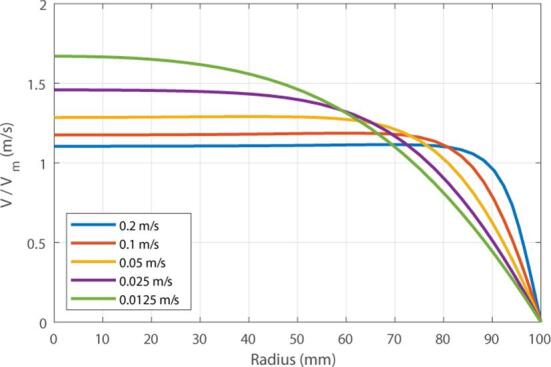
Evolution of the relative pulp flow velocity magnitude.

In the following, for simplicity, only the eucalyptus pulp is considered but the same tendencies can be observed for pine pulp. In Fig. 7, the evolution of the mean flow profile and radial drag force with respect to the inlet mean velocity is presented. As flow velocity increases the viscosity decreases inducing a modification of the flow profile along the radius with a transition from the laminar regime, exhibiting a quadratic profile below 0.025 m/s, to the turbulent regime above 0.05 m/s.

Thus, low velocities are more interesting for acoustophoresis in the case where the drag force is predominant. In addition, as presented in the introduction and observed in Fig. 7, at velocity below 0.05m/s , the pulp flow behaves like a laminar fluid with a boundary layer at the pipe edges. By working at low velocities, it is possible to avoid the inconveniences caused by the turbulence of the pulp flow, especially the turbulent and radial movements of the particles, thus facilitating the manipulation of the pulp fibers by the ultrasound field while increasing the exposure time to ultrasound.

Based on Eq. (4), the pulp consistency can be considered as a change of viscosity μ . At low consistency (i.e. below 0.1 %), a very low viscosity μ<5Pa.s is observed, and the pulp flow can be approximated as laminar as shown in Fig. 7. This behavior is observed for consistency below 1 %. For consistencies above 1 %, high viscosities above μ>10Pa.s are present. The turbulent regime is then observed, inducing a uniform flow velocity through the pipe. As observed previously, this increase of consistency can be interpreted as an increase of the fluid viscosity. Consequently, the drag force is increased by a factor of 100 between 0.1 % and 6 %, and the acoustic damping is increased as reported in [18]. Hence, it has been observed that the maximal efficiency can be obtained for consistencies below 1 %.

### 4.4 Feasibility of acoustophoresis for pulp concentration

**Fig. 8:**
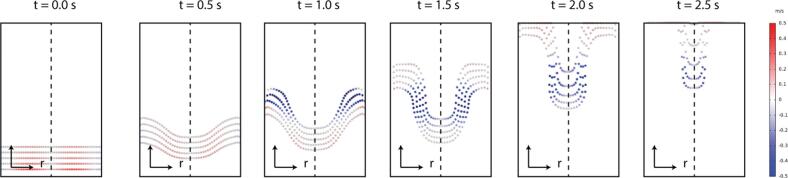
Evolution of the pulp particle position and radial velocity over time between 0 and 2.5 s.

In order to determine if the acoustophoresis is a valid option for paper pulp concentration, the particle deviation is derived from the drag and radiation force fields. From the parametric study conducted previously, optimal parameters are derived. A pulp consistency of 1 % for eucalyptus is selected as representative of the industrial process and the evaluation of pulp concentration is performed using particle tracking on the COMSOL multiphysics model. These particles are released at the velocity $u_{0}+u_{2}$ in a zone where z is smaller than 0.2 m. The particle velocity follows the pulp velocity, since the velocity $u_{2}$ induced by the acoustic streaming, is negligible in this zone. In Fig. 8, the evolution of particles subjected to acoustophoresis is presented at different time steps representative of the process. The particle radial velocity is presented using a colormap. This velocity is between -0.5m/s and 0.5m/s .

As shown in the first timeframes of Fig. 8, before the particles reach the zone where the radial velocity $u_{2}$ is maximal, the drag force is negligible. Thus, the particles are not deflected towards the center of the pipe but an axial force is slowing down the particle at the center of the pipe. From t=1.0 s onwards, a change in the trajectory of the first particles can be observed in Fig.8. It is at this point that the first particles are exposed to the high intensity area of drag force. The radial velocity of the particles becomes negative, which is representative of a movement towards the center of the pipe. After 2.5 s the acoustic streaming no longer has any real impact on the particle deflection. Moreover, the radiation force has no significant impact on the trajectory of the particles and no concentration around pressure nodes are observed.

Based on this trajectory, the deviation of a line of particles at the end of the modeled region can be extracted as a function of their initial position. The overall shape of the particle deviation function is different from the radial drag force plot in front of the transducer, since the pulp velocity flow increases the deflection of the particles close to the pipe wall (r=100mm). Based on the proposed configuration, a maximum deflection of 10 mm is observed for this configuration, allowing derivation of the concentration gain achievable with these parameters. For this purpose, the number of particles arriving on either side of a virtual separator is estimated. This allows the outgoing flow to be divided into two parts: a central flow concentrated in particles and a peripheral flow non-concentrated in particles. In this situation, if a separator is located at a radius of 75 mm, a concentration gain of 15% is obtained, such that the central flow has a consistency of 1.15 % and the peripheral flow has a concentration of 0.85 % for an incident consistency of 1 %. Acoustophoresis is thus realistic in this configuration but will require an iterative process of at least five transducer rows in order to achieve a concentration gain of 100 % (from 3 % to 6 %).

Other configurations could also be envisioned for axisymmetric pulp concentration. Indeed, based on the numerical observations, the drag force which is predominant in the present process, is proportional to the generated pressure field. For this reason, a number of narrow channels could be used in order to reduce the propagation distance and thus increase the pressure field. However, in practice, the viscosity and possible aggregation of the pulp limits the pipe diameter to 50 mm, such that standing waves will be observed above 30 kHz, reducing the efficiency of the process as presented in Fig. 6. Moreover, reducing the pipe diameter limits the transducer footprint and may thus impair the axial symmetry of the problem. Other design options concerning the inner pipe could also be considered. In the present study, the choice of a large inner pipe (15 cm diameter) is preferred in order to collect clear water at the outside pipe. However, for certain applications, smaller inner pipe diameters could be used to collect more concentrated pulp at the center, but this would require recirculation of the outer pipe flow.

## 5 Experimental validation

### 5.1 Experimental setup

**Fig. 9:**
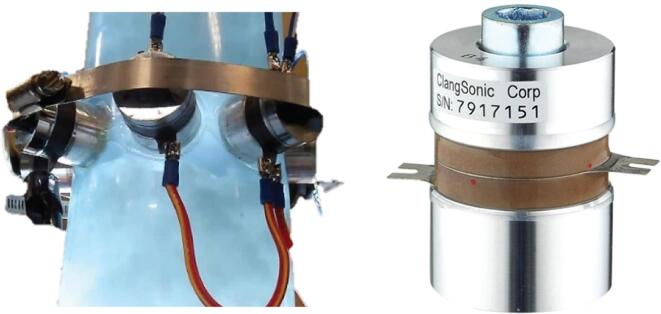
Axisymmetric ultrasonic chamber (left) and used transducers (right).

In order to validate the numerical results obtained in the previous section and determine if the proposed axisymmetric configuration is viable, an experimental test bench has been designed and manufactured at Université de Sherbrooke and is presented in Fig. 9. A 20 cm diameter acrylic pipe is instrumented using 8 identical 50 W transducers (Clangsonic - CN12538-40HB P8) with a resonance frequency of 125 kHz (first compression mode). The transducers are mounted using epoxy and a collar is added in order to apply a static pressure on the transducer ring. The transducers are connected in parallel to a power amplifier (E&I Electronics – 1040 L – 400 W) with a gain of 55 dB that is driven by an Agilent 33120A function generator used in sinusoidal mode.

**Fig. 10:**
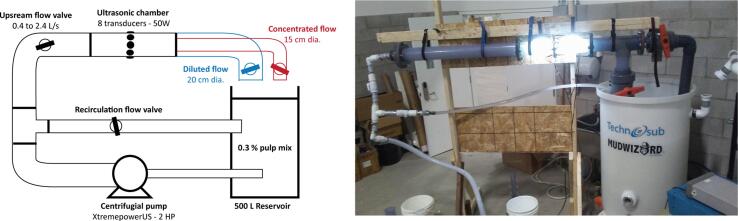
Description of the recirculation setup (left) and photograph of the experiment (right).

The chamber is then mounted on a recirculation circuit as presented in Fig. 10. For this purpose, a 2.0 HP self-discharge center discharge pump (XtremepowerUS 2.0 HP) is used for recirculation of a water/pulp mix. The upstream flow rate is controlled between 0.4 L/s and 2.4 L/S using a recirculation circuit and ball valves. The upstream flow is stabilized by ensuring a distance of 1 m before entering the ultrasonic chamber. This ensures a flow velocity ranging between 0.01 m/s and 0.07 m/s. In the downstream flow, a concentric separator has been manufactured in order to separate the concentrated flow for the diluted flow using a 15 cm diameter pipe inside a 20 cm diameter pipe. The pressures and flowrates at both flows are controlled using ball valves in order to limit air intake in the ultrasonic chamber. A total of 1.25 kg of softwood pulp has been diluted in 425 L of water in order to reach a concentration of 0.3 % in the upstream flow.

The concentration at both diluted and concentrated flows is measured using a controlled volume of 1 L and by measuring the height of fiber after degassing and decantation over two days. Three flowrates have been tested in order to derive the concentration factor, namely 0.4 L/s, 1.5 L/s and 2.4 L/s and for each flowrate, one samples is collected every minute for a total of 3 samples for statistical purpose.

### 5.2 Experimental results

**Fig. 11:**
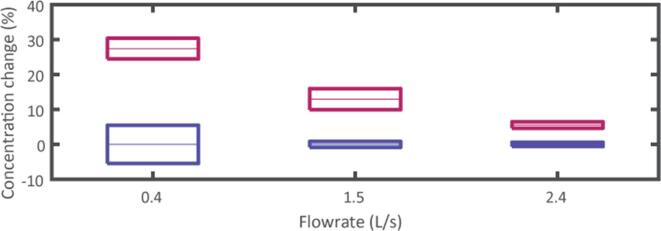
Relative concentration (in red) with respect to the reference configuration (without ultrasound – in blue) for three flowrates. (For interpretation of the references to colour in this figure legend, the reader is referred to the web version of this article.)

Fig. 11 presents the results obtained for the 3 considered flowrates. In each case, the reference measurements (in blue) compared to the measured concentrated flow (red). The results are normalized with respect to the mean reference value and presented as boxplots representing the mean value and standard deviations based on the simple statistical analysis of the 3 samples. In Fig. 11, an increase of pulp concentration is observed in all the considered cases and a concentration gain up to 30 % is reached when considering a flowrate of 0.4 L/s. When increasing the flowrate, the concentration gain decreases, reaching 5 % at 2.4 L/s. The variability of the results at the flowrate of 0.4 L/s can be attributed to gravity effects that occur and are responsible for a decantation effect that is responsible for local sedimentation prior to flow separation. This effect is however, not observed above 1.5 L/s, explaining the strong repeatability at higher flowrates.

Visually, no circumferential flows are observed when applying ultrasound waves, but rotational flows (local recirculation and pulp ejection) appear around the transducers with an amplitude varying across the circumference of the pipe. This can be attributed to the imperfections in the transducer mounting and resonance frequency that impair the axisymmetric assumption, and are thus responsible for discrepancies in the acoustic velocity field. However, the experimental observations are still in good agreement with the numerical predictions, such that the proposed method seems appropriate for low consistency pulp concentration. Stabilization of the process is obtained after maximum 15 s, and the tests have been conducted for longer exposure time (up to 5 min) in order to verify the robustness and repeatability of the process. After 5 min of ultrasound application, the performances are stable, but a local heating of the ultrasonic chamber has been observed, and is responsible for a local deformation of the PVC pipe. Thus, for industrial application, metallic pipes and temperature resistant bonding techniques should be considered.

## 6 Conclusion

In the present study, an axisymmetric numerical model combining acoustophoresis with a turbulent flow of particles in a viscous fluid for paper pulp concentration in a pipe is derived. The acoustic levitation model highlights the duality between the acoustic radiation force and the drag force. Due to the high viscosity of the pulp flow, the drag force is dominant. Based on this prediction, an axisymmetric configuration that deflects the pulp fibers towards the center of a pipe is proposed. A parametric study has been conducted allowing to derive the influence of the transducer and pulp flow parameters on the drag and radiation forces. To have important particle exposure time at the maximum sound pressure zone, it is necessary to have a low velocity flow. The particles are then deflected towards the center of our pipe and their position is determined in order to derive the efficiency of the process. Depending on the parameters used, the deviation reaches up to 10 mm, and can be assimilated to a concentration gain of 15 %. The experimental validation has demonstrated the feasibility of pulp concentration up to 30 % at low flowrate for low consistency mixtures. Ongoing studies concern the industrial application and large-scale feasibility of this process for the paper industry.

### CRediT authorship contribution statement

**Romain Le Magueresse:** Conceptualization, Methodology, Software, Writing – original draft. **Tamara Krpic:** Conceptualization, Methodology, Writing – review & editing. **Maxime Bilodeau:** Writing – review & editing, Visualization. **Robert Schiavi:** Conceptualization, Methodology, Validation, Resources, Writing – review & editing, Supervision, Project administration. **Pierre Gelinas:** Writing – review & editing, Funding acquisition. **Nicolas Quaegebeur:** Supervision, Project administration, Conceptualization, Methodology, Validation, Funding acquisition.

## Declaration of Competing Interest

The authors declare that they have no known competing financial interests or personal relationships that could have appeared to influence the work reported in this paper.
